# Patient-reported symptoms before adjuvant locoregional radiotherapy for breast cancer: triple-negative histology impacts the symptom burden

**DOI:** 10.1007/s00066-024-02224-8

**Published:** 2024-03-26

**Authors:** Carsten Nieder, Silje K. Johnsen, Annette M. Winther, Bård Mannsåker

**Affiliations:** 1 Department of Oncology and Palliative Medicine, Nordland Hospital Trust, 8092 Bodø, Norway; 2 Department of Clinical Medicine, Faculty of Health Sciences, UiT—The Arctic University of Norway, Tromsø, Norway

**Keywords:** Postoperative radiation treatment, Edmonton Symptom Assessment System, Patient-reported symptoms, Pathological complete remission, Hormone receptor negative disease

## Abstract

**Background:**

Multimodal breast cancer treatment may cause side effects reflected in patient-reported outcomes and/or symptom scores at the time of treatment planning for adjuvant radiotherapy. In our department, all patients have been assessed with the Edmonton Symptom Assessment System (ESAS; a questionnaire addressing 11 major symptoms and wellbeing on a numeric scale of 0–10) at the time of treatment planning since 2016. In this study, we analyzed ESAS symptom severity before locoregional radiotherapy.

**Patients and methods:**

Retrospective review of 132 patients treated between 2016 and 2021 (all comers in breast-conserving or post-mastectomy settings, different radiotherapy fractionations) was performed. All ESAS items and the ESAS point sum were analyzed to identify subgroups with higher symptom burden and thus need for additional care measures.

**Results:**

The biggest patient-reported issues were fatigue, pain, and sleep problems. Patients with triple negative breast cancer reported a higher symptom burden (mean 30 versus 20, *p* = 0.038). Patients assigned to adjuvant endocrine therapy had the lowest point sum (mean 18), followed by those on Her-2-targeting agents without chemotherapy (mean 19), those on chemotherapy with or without other drugs (mean 26), and those without systemic therapy (mean 41), *p* = 0.007. Those with pathologic complete response after neoadjuvant treatment had significantly lower anxiety scores (mean 0.7 versus 1.8, *p* = 0.03) and a trend towards lower depression scores, *p* = 0.09.

**Conclusion:**

Different surgical strategies, age, and body mass index did not impact on ESAS scores, while the type of adjuvant systemic therapy did. The effect of previous neoadjuvant treatment and unfavorable tumor biology (triple negative) emerged as important factors associated with symptom burden, albeit in different domains. ESAS data may facilitate identification of patients who should be considered for additional supportive measures to alleviate specific symptoms.

## Introduction

During decades of successful adjuvant radiotherapy for breast cancer, reduction of acute and late side effects such as skin reactions and pneumonitis has been an important topic of research, aiming at a continuous improvement of the therapeutic ratio [1, 2]. More recently, health-related quality of life (QoL) and patient-reported outcomes (PROs) have been evaluated as well, because physician-scored side effects are unable to mirror the complex patient experience during a typically multimodal sequence of different treatments [3]. Preceding treatment such as neoadjuvant drugs and definitive surgery (breast-conserving or mastectomy with different approaches regarding axillary lymph nodes) may cause symptoms that are still present when patients move forward to the adjuvant phase, where new drugs and/or radiotherapy can impact QoL and PROs [4]. A wide range of symptoms including but not limited to pain, fatigue, anxiety, and sleep disturbance are reported by many patients before they start with adjuvant radiotherapy.

Many different instruments have been used to evaluate QoL and PROs [5]. Currently, mobile apps and other digital solutions are gaining increasing importance [6, 7]. The Edmonton Symptom Assessment System (ESAS), originally developed in the palliative care setting [8], has occasionally been employed in curatively treated patients with breast cancer [9–14]. ESAS is a short, one-sheet questionnaire addressing major symptoms and wellbeing on a numeric scale of 0–10, which can easily be integrated into routine workflow in radiation oncology departments [15]. The radiotherapy facility at Nordland Hospital started screening of all palliatively irradiated patients with the ESAS tool in 2012, following the routine procedure that already had been in place for outpatients receiving systemic anticancer therapy for several years. From 2016, patients seen for treatment planning of adjuvant radiotherapy for breast cancer also were asked to provide a symptom assessment, which may facilitate initiation of measures that contribute to better symptom control. Given that patients with more advanced disease who received more intense preceding treatment may be more likely to experience toxicity and related symptoms, we limited this initial study of ESAS data in our institution’s breast cancer patients to those referred to locoregional radiotherapy. Potential correlations between patient-related parameters, e.g., age or body mass index (BMI), comorbidity, disease-related parameters, and treatment parameters on one hand and pre-radiotherapy symptom severity on the other were assessed.

## Materials and methods

We performed a retrospective analysis of 132 unselected, consecutive female patients who started locoregional adjuvant radiotherapy at our hospital during the time period 2016–2021. Radiotherapy was administered after 3D planning, mostly with daily 2‑Gy fractions with or without sequential boost in deep-inspiration breath-hold. Hybrid intensity-modulated techniques were employed on an individual basis. The same was true for hypofractionation (15 fractions of 2.67 Gy), e.g., in elderly patients. The ESAS tool was administered by a registered oncology nurse immediately before radiation oncologist consultation and computed tomography imaging for treatment planning approximately 1 week before radiotherapy. All medical records were available in the hospital’s electronic patient record (EPR) system. Statistical analysis was performed with IBM SPSS Statistics 29 (IBM Corp., Armonk, NY, USA). In addition to relevant ESAS items of interest (continuous variables expressed as mean with standard deviations [SD]), we analyzed a large number of categorical baseline variables (dichotomized present/absent or categorized by quartiles or treatment groups). Analysis of variance (ANOVA) tables were employed for inter-group comparisons. A *p*-value ≤ 0.05 was considered statistically significant.

## Results

The mean age was 59 years (SD 13), range 24–88 years. The mean BMI was 27.7 kgm^-2^ (SD 5), range 18–45 kgm^-2^. Most patients had pT1 or 2 node-positive (N1) disease. Table 1 shows additional baseline characteristics and Table 2 shows the ESAS data. The biggest patient-reported issues were fatigue, pain (while moving), and sleep problems. To reduce the likelihood of spurious findings from multiple testing, several items (appetite, nausea, constipation, dyspnea, dry mouth) were not carried forward to further individual analyses, unless special subgroups of interest appeared in the first round where all items were employed to create the point sum. In addition, the patient-reported item “overall wellbeing” possibly integrates all the different aspects, and was analyzed as a secondary outcome of interest.

**Table 1:** Baseline characteristics before adjuvant radiotherapy in 132 female patients

Variable	*N*	%
Screening detected	28	21
*Pathological stage*		
pT0	19	14
pT1	53	40
pT2	49	37
pT3	5	4
pT4	6	5
pN0	34	26
pN1	71	54
pN2	17	13
pN3	2	2
Microscopic nodal disease only	8	6
*Histology*		
Ductal carcinoma	113	86
Lobular carcinoma	12	9
Both	2	2
Others	5	4
*Histological grade*		
G1	24	18
G2	57	43
G3	29	22
Uncertain after NATx^a^	22	17
*Receptor status*		
Estrogen negative	32	24
Progesterone negative	54	41
Triple negative	17	13
Her2 positive	26	20
*Others*		
*BRCA* mutation	4	3
Premenopausal^b^	26	20
Perimenopausal	13	10
Postmenopausal	90	68
Diabetes mellitus	8	6
Autoimmune disease	15	11
Active smoker^b^	12	9
*Treatment*		
Neoadjuvant systemic therapy	65	49
Mastectomy	75	57
Breast conservation	57	43
Re-excision (second breast surgery)	11	8
Axillary dissection	69	52
Sentinel node biopsy	63	48
Hypofractionated radiotherapy	9	7
Tumor bed boost	20	15
Adjuvant systemic therapy	126	95

^a^NATx: neoadjuvant systemic therapy

^b^Missing information in some cases

**Table 2:** Edmonton Symptom Assessment System (ESAS) before adjuvant radiotherapy in 132 female patients

Item	Median	Mean	Range
Pain (not moving)	0	1.65	0–9
Pain (while moving)	2	2.38	0–10
Fatigue	3	2.95	0–10
Nausea	0	0.53	0–8
Dyspnea	0	1.14	0–10
Dry mouth	1	2.52	0–10
Appetite	0	1.43	0–8
Constipation	0	0.96	0–10
Anxiety/restlessness	1	1.67	0–10
Sleep	2	2.65	0–10
Sadness/depression	0	1.36	0–10
Overall wellbeing	2	2.35	0–10
Point sum	19.5	21.50	0–86

All the different parameters displayed in Table 1 were analyzed for correlations with the ESAS point sum. Only two significant correlations were identified: patients with triple-negative breast cancer reported a higher symptom burden (mean 30 versus 20, *p* = 0.038). Patients assigned to adjuvant endocrine therapy (*n* = 72), which had typically not yet been started at the time of ESAS scoring, had the lowest point sum (mean 18), followed by those on Her-2-targeting agents without chemotherapy (*n* = 14, mean 19), those on chemotherapy with or without other drugs (*n* = 40, mean 26), and those without systemic therapy (*n* = 5, mean 41), *p* = 0.007. It should be noted that patients without adjuvant systemic therapy had triple-negative breast cancer. However, some patients with triple-negative disease received adjuvant chemotherapy.

The fact that patients with triple-negative disease had higher mean point sums resulted from three individual items: fatigue (mean 4.1 versus 2.8, *p* = 0.05), pain (not moving; mean 3.1 versus 1.4, *p* = 0.002), and pain (while moving; mean 4.1 versus 2.1, *p* = 0.004). Regarding adjuvant systemic therapy, the same three ESAS items were the main drivers of higher symptom burden. Mean fatigue scores were 2.2 (endocrine), 3.6 (chemotherapy), 4.0 (Her-2), and 6.0 (no adjuvant drugs), *p* < 0.001. Mean pain scores (not moving) were 1.2 (endocrine), 2.1 (chemotherapy), 2.0 (Her-2), and 4.4 (no adjuvant drugs), *p* = 0.003. The corresponding figures for pain in activity were 2.2 (endocrine), 3.6 (chemotherapy), 2.7 (Her-2), and 6.8 (no adjuvant drugs), *p* < 0.001.

Additional differences were observed regarding dry mouth (mean 2.0 after adjuvant chemotherapy, 3.5 in the Her‑2 group, and 2.5 in both other groups, *p* = 0.045). Patients not receiving adjuvant systemic therapy had reduced appetite (mean 2.8 versus 1–2 in all other groups, *p* = 0.08).

After the primary analysis of the ESAS point sum, a second analysis was run with overall wellbeing as the endpoint, without identifying any significant correlations (all *p*-values > 0.1).

Finally, we were interested in the prognostically favorable group with stage ypT0, i.e., pathologic complete response at surgery due to neoadjuvant treatment. These patients had significantly lower mean anxiety scores (0.7 versus 1.8, *p* = 0.03) and a trend towards lower depression scores (mean 0.6 versus 1.5, *p* = 0.09) compared to all other patients (T1–4 with or without neoadjuvant treatment; *n* = 19 versus 113).

## Discussion

The present study addressed patient-reported symptoms in an unselected real-world cohort, which included patients with different disease characteristics and treatment pathways. At the time of treatment planning, 132 patients scored their symptoms with the ESAS tool. Canadian researchers have also published several studies on ESAS and curative breast cancer treatment. Chow et al. reported no statistical difference in ESAS scores for mastectomy and lumpectomy patients [9], a finding confirmed in our study. Neither re-resection nor the extent of surgical axillary treatment were associated with ESAS scores in the present cohort. Barbera et al. evaluated the impact of screening with ESAS on emergency department (ED) visit rates in women with breast cancer receiving adjuvant chemotherapy [10]. Interestingly, screening with ESAS was associated with decreased ED visits. Chow et al. performed a longitudinal radiotherapy study including both ESAS and QoL [11]. Of the ESAS symptoms identified as significant predictors of QoL, pain, fatigue, and anxiety correlated with overall wellbeing at all timepoints. Given that such data provide reasons to believe that overall wellbeing to some degree integrates many of the individual items, our own data failed to show that overall wellbeing is of major importance as a global symptom burden criterion. We would rather advocate for employment of the ESAS point sum when researchers try to summarize the symptom burden. While the latter may facilitate statistical comparisons, clinicians need to look at the complete picture, trying to identify the individual problems of each patient in order to increase care-associated satisfaction [16].

At our department, ESAS assessment during radiotherapy and follow-up has not been performed. Lam et al. reported longitudinal data showing that patient-reported pain associated with breast irradiation peaked 1 week after treatment completion [12]. Younger patients (40–49 or 50–59 years of age) reported significantly more overall pain and breast pain compared with patients ≥ 60 years of age. Our own study did not specifically address different locations of pain, but rather collected overall pain data only. In principle, the latter may be influenced by comorbidities and age as well as medications. It should also be noticed that the Canadian studies were larger than the present one. Behroozian et al. compared breast cancer patients with and without regional nodal irradiation and included 781 patients in the longitudinal analysis [13]. Baseline symptom reporting was similar between cohorts. Across all timepoints, differences in outcomes between cohorts were minimal, except for lack of appetite (*p* = 0.03), which was significantly aggravated in patients treated with regional nodal irradiation.

A major finding from the present study was the impact of adjuvant systemic treatment, which is connected to tumor type, because specific drugs are restricted to patients with Her-2-positive disease, while triple-negative histology restricts adjuvant options in a broader sense, especially in the earlier years of this study before capecitabine was introduced. Akkila et al. studied patients treated between February 2018 and September 2020 [14]. They compared baseline scores between adjuvant and neoadjuvant chemotherapy patients (*n* = 338). Comparison of baseline ESAS scores revealed that patients who received adjuvant chemotherapy were more likely to report higher scores, reflecting higher symptom burden, compared to patients receiving neoadjuvant chemotherapy, including fatigue (*p* = 0.005), lack of appetite (*p* = 0.0005), and dyspnea (*p* < 0.0001). So far, no other studies have examined all the baseline and treatment parameters we were able to include. Our results suggest that patients with triple-negative disease may require particular attention. Previous research showed that women with triple-negative tumors had worse QoL compared to those with non-triple-negative tumors [17]. Recently, a different study with less-well studied endpoints showed that chemotherapy, triple-negative tumor, reconstructive surgery, number of outpatient visits, and income were associated with prolonged sick leave [18].

The present study also showed a certain impact of pathologic complete response at surgery due to neoadjuvant treatment on some ESAS items. Exceedingly chemosensitive patients had significantly lower mean anxiety scores (*p* = 0.03) and a trend towards lower depression scores (*p* = 0.09). Awareness of the excellent prognosis would explain why these patients were less worried in the adjuvant setting. Interestingly, triple-negative patients were not even close to reporting significantly higher anxiety or depression scores than their non-triple-negative counterparts. This fact would argue against prognostic worries as a reason for a mainly fatigue- and pain-driven higher ESAS sum. Additional research is needed to fully elucidate our findings.

When interpreting the present results, the following limitations must be acknowledged: our study cohort was comprised of Norwegian-speaking patients covered by the national publicly funded health care system. In a more diverse setting, socioeconomic factors may interfere with QoL and PROs. Since the study size and consequently statistical power were limited, we may have overlooked additional correlations that a larger study could have revealed. Longitudinal data from various timepoints during radiotherapy may provide additional information when trying to provide comprehensive, individualized supportive measures such as physical exercise, physiotherapy, psycho-oncology referral, rehabilitation, and others [19, 20], aiming at high rates of treatment completion, better QoL and role-functioning, and minimum interference of breast cancer treatment with survivors’ daily life.

## References

[1] Haussmann J, Budach W, Corradini S, Krug D, Jazmati D, Tamaskovics B, Bölke E, Pedotoa A, Kammers K, Matuschek C (2023). Comparison of adverse events in partial- or whole breast radiotherapy: investigation of cosmesis, toxicities and quality of life in a meta-analysis of randomized trials. Radiat Oncol.

[2] Mangesius J, Minasch D, Fink K, Nevinny-Stickel M, Lukas P, Ganswindt U, Seppi T (2023). Systematic risk analysis of radiation pneumonitis in breast cancer: role of cotreatment with chemo-, endocrine, and targeted therapy. Strahlenther Onkol.

[3] Hauth F, De-Colle C, Weidner N, Heinrich V, Zips D, Gani C (2021). Quality of life and fatigue before and after radiotherapy in breast cancer patients. Strahlenther Onkol.

[4] Piroth MD, Draia S, Jawad JA, Piefke M (2022). Anxiety and depression in patients with breast cancer undergoing radiotherapy: the role of intelligence, life history, and social support-preliminary results from a monocentric analysis. Strahlenther Onkol.

[5] Remick JS, Kowalski E, Samanta S, Choi S, Palmer JD, Mishra MV (2020). Health-related quality of life and patient-reported outcomes in radiation oncology clinical trials. Curr Treat Options Oncol.

[6] Schunn FA, El Shafie RA, Kronsteiner D, Sauer LD, Kudak A, Bougatf N, Oetzel D, Krämer A, Regnery S, Machmer T, Debus J, Nicolay NH (2024). Oncologic treatment support via a dedicated mobile app: a prospective feasibility evaluation (OPTIMISE-1). Strahlenther Onkol.

[7] Janssen S, El Shafie RA, Grohmann M, Knippen S, Putora PM, Beck M, Baehr A, Clemens P, Stefanowicz S, Rades D, Becker JN, Fahlbusch FB (2024). Survey in radiation oncology departments in Germany, Austria, and Switzerland: state of digitalization by 2023. Strahlenther Onkol.

[8] Bruera E, Kuehn N, Miller MJ, Selmser P, Macmillan K (1991). The Edmonton Symptom Assessment System (ESAS): a simple method for the assessment of palliative care patients. J Palliat Care.

[9] Chow R, Pulenzas N, Zhang L, Ecclestone C, Leahey A, Hamer J, DeAngelis C, Bedard G, McDonald R, Bhatia A, Ellis J, Rakovitch E, Vuong S, Chow E, Verma S (2016). Quality of life and symptom burden in patients with breast cancer treated with mastectomy and lumpectomy. Support Care Cancer.

[10] Barbera L, Sutradhar R, Howell D, Sussman J, Seow H, Dudgeon D, Atzema C, Earle C, Husain A, Liu Y, Krzyzanowska MK (2015). Does routine symptom screening with ESAS decrease ED visits in breast cancer patients undergoing adjuvant chemotherapy?. Support Care Cancer.

[11] Chow S, Wan BA, Pidduck W, Zhang L, DeAngelis C, Chan S, Yee C, Drost L, Leung E, Sousa P, Lewis D, Lam H, Chow R, Lock M, Chow E (2019). Symptoms predictive of overall quality of life using the Edmonton symptom assessment scale in breast cancer patients receiving radiotherapy. Clin Breast Cancer.

[12] Lam E, Wong G, Zhang L, Drost L, Karam I, Yee C, McCurdy-Franks E, Razvi Y, Ariello K, Wan BA, Nolen A, Wang K, DeAngelis C, Chow E (2021). Self-reported pain in breast cancer patients receiving adjuvant radiotherapy. Support Care Cancer.

[13] Behroozian T, Milton L, Zhang L, Lou J, Shariati S, Karam I, Chow E (2023). A comparison of acute patient-reported outcomes in breast cancer patients with and without regional nodal irradiation using the ESAS and PRFS tool. Support Care Cancer.

[14] Akkila S, Shariati S, Milton L, Behroozian T, Zhang L, Lou J, Lam E, Wong G, Karam I, Chow E (2023). Comparison of neoadjuvant versus adjuvant chemotherapy for breast cancer patients prior to receiving radiation therapy using Edmonton Symptom assessment system (ESAS) scores. Support Care Cancer.

[15] Nieder C, Kämpe TA, Pawinski A, Dalhaug A (2018). Patient-reported symptoms before palliative radiotherapy predict survival differences. Strahlenther Onkol.

[16] Fabian A, Rühle A, Domschikowski J, Trommer M, Wegen S, Becker JN, Wurschi G, Boeke S, Sonnhoff M, Fink CA, Käsmann L, Schneider M, Bockelmann E, Treppner M, Mehnert-Theuerkauf A, Nicolay NH, Krug D, Young DEGRO Group (2024). Satisfaction with radiotherapy care among cancer patients treated in Germany—secondary analysis of a large multicenter study. Strahlenther Onkol.

[17] Vadaparampil ST, Christie J, Donovan KA, Kim J, Augusto B, Kasting ML, Holt CL, Ashing K, Halbert CH, Pal T (2017). Health-related quality of life in Black breast cancer survivors with and without triple-negative breast cancer (TNBC). Breast Cancer Res Treat.

[18] Leskelä RL, Haavisto I, Pennanen P, Lahelma M, Mattson J, Poikonen-Saksela P (2023). Predictive factors for prolonged sick leave in breast cancer patients treated with adjuvant therapies: a retrospective registry study. Acta Oncol.

[19] Gjerset GM, Skaali T, Seland M, Thorsen L (2023). Health-related quality of life, fatigue, level of physical activity, and physical capacity before and after an outpatient rehabilitation program for women within working age treated for breast cancer. J Cancer Educ.

[20] Medeiros Torres D, Koifman JR, da Silva SS (2022). Impact on fatigue of different types of physical exercise during adjuvant chemotherapy and radiotherapy in breast cancer: systematic review and meta-analysis. Support Care Cancer.

